# Preparing sequencing grade RNAs from a small number of FACS-sorted larvae macrophages isolated from enzyme free dissociated zebrafish larvae

**DOI:** 10.1016/j.mex.2022.101651

**Published:** 2022-03-09

**Authors:** Christina Begon-Pescia, Stéphanie Boireau, Myriam Boyer-Clavel, Georges Lutfalla, Mai Nguyen-Chi

**Affiliations:** a CNRS, LPHI, Universite Montpellier, Montpellier, France; b Montpellier Ressources Imagerie, Biocampus, CNRS, INSERM, Universite Montpellier, Montpellier, France

**Keywords:** Zebrafish larva, Macrophages dissociation, FACS-sorted, RNA quality

## Abstract

Macrophages are phagocytic cells from the innate immune system that are critical for tissue homeostasis and form the first line of host defense against invading pathogens. The zebrafish larva is an exquisite model to decipher the transcriptional response of macrophages after injury. We used a macrophage reporter line in which an *mfap4* promoter drives the expression of a farnesylated mCherry fluorescent protein to label macrophages and we performed tissue dissociation, cell isolation by Fluorescence Activated Cell sorting and RNA preparation. The two bottlenecks are (i) the dissociation of the embryos that often relies on cell suspension steps that alter the activation status of immune cells, and (ii) obtaining high RNA integrity for gene expression analysis from a small number of isolated macrophages. Here, we describe (i) the dissociation of cells from whole *Tg(mfap4:mCherry-F)* zebrafish larvae using an enzyme-free and osmotically controlled buffer, (ii) the sorting of fluorescent macrophages by FACS and (iii) the preparation of high quality RNAs for meaningful gene expression analysis from a small number of isolated macrophages.

• An optimized protocol in 5 steps to extract high quality RNAs from zebrafish macrophages.
• A cell dissociation method using an enzyme-free and osmotically controlled buffer to prevent the alteration of macrophage activation status and limit cell mortality.
• Production of high integrity RNAs from a small number of isolated macrophages.

## Graphical abstract

**Figure:**
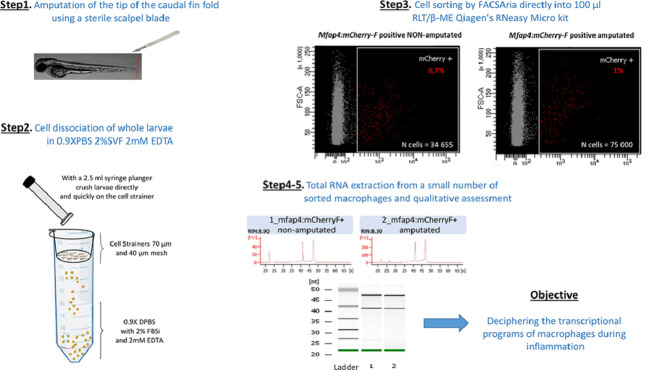

Specification table

**Table:** 

Subject area;	Immunology and Microbiology
More specific subject area;	Establishment of immunity and inflammation, zebrafish
Protocol name;	A good RNA integrity from a small number of FACS-sorted macrophages for gene expression analysis
Reagents/tools;	All reagents and tools are described in description of protocol
Experimental design;	This protocol was optimized based on the Nguyen-Chi et al., publication [6,5]. The protocol is divided in five steps: (1) Larvae caudal fin amputation (2) Enzyme-free cell dissociation using an osmotically controlled buffer for the sorting (3) macrophage cell sorting by the FACSAria™ IIu (4) Total RNA extraction from a small number of sorted macrophages (5) Qualitative assessment of total RNA.
Trial registration;	This study does not include Clinical trial.
Ethics;	All animal experiments described in the present study were conducted at the University of Montpellier according to European Union guidelines for handling of laboratory animals (http://ec.europa.eu/environment/chemicals/lab_animals/home_en.htm and were approved by the Direction Sanitaire et Vétérinaire de l'Hérault and Comité d'Ethique pour l'Expérimentation Animale under references CEEA-LR-13007 and 2016061511212601. Larvae are used at the stage of 3 days post-fertilization.
Value of the Protocol;	This protocol is useful for all those interested in preparing RNAs from small numbers of FACS-sorted zebrafish macrophages since the less material, the more difficult it is to obtain high quality RNAs compatible with transcriptomics analysis. Contrarily to most protocols to dissociate larvae that use enzymes (collagenase, trypsin) [4,2,1], we use a protocol that is enzyme free and osmotically controlled buffer, because we have shown that the use of trypsin to dissociate larvae induces high level expression of pro-inflammatory genes [5].
Declaration of interests;	The authors declare that they have no known competing financial interests or personal relationships that could have appeared to influence the work reported in this paper.

## Protocol details

### Zebrafish line and maintenance

Zebrafish (*Danio rerio*) maintenance, staging and husbandry were performed in the fish facility of LPHI (University of Montpellier) as described [8]. Males and females were kept in 3.5-L polycarbonate tanks connected to a recirculating Tecniplast system in following condition: 0.4% salinity/ 400 µS conductivity, temperature of 28 °C and a 12:12-h light: dark cycle. The fish were fed twice per day with Skreting GEMMA Micro 500. Embryos were obtained from pairs of adult fish by natural spawning. Embryos were collected and reared at 28 °C in 10-cm petri dishes (60 embryos/dish) containing about 25 ml of zebrafish water supplemented with methylene blue 0.1% (w/v), according to standard condition [3]. Until the desired stage, the zebrafish water is changed every day and for the experiments, larvae were staged according to Kimmel et al., and used from 3 to 4 days post-fertilization (dpf). Experiments were performed using AB wild-type zebrafish and *Tg*(*mfap4: mCherry-F)*ump6Tg animals referred here as *Tg(mfap4:mCherry-F)* [9].

#### Caudal fin fold amputation (step1)

*Equipment*

- Sterile carbon steel surgical blades size 10 (Scalpel Paramount™)
- Petri dish 100 × 15 (Corning Gosselin™ # BP93B-102)
- Graduated transfer pipet 3.9 ml (Samco™ # 225)
- Stereomicroscope Motic # SMZ-171 / # SMZ-168 or equivalent
- Generic laboratory equipment

*Materials*

- Living zebrafish *Tg(mfap4:mCherry-F)* larvae: 360 larvae for amputated condition and 360 larvae for non amputated condition at 3 days post-fertilization (dpf)
- Living zebrafish wild-type AB larvae: 200 larvae at 3 days post-fertilization (dpf)

*Reagents*

- Stock Anaesthesia Tricaine solution: 400 mg (Ethyl 3-aminobenzoate methanesulfonate, MS-222 Sigma #A5040) added to 100 ml of osmosis water and buffered by addition of 2.1 ml 1 M Tris pH 9. The Tricaine stock solution is aliquoted and stored at −20 °C
- Zebrafish water pH 7–8. For 1L: 1.5 ml of Instant Ocean Sea Salt mixture stock solution (40 g/L), 40 µl 10 N NaOH and 300 µl methylene blue stock solution (sigma # M9140)
- Sodium Hydroxide (NaOH _MW=40.00_) stock solution, 10 N equal to 10 M (For 100 ml: 40 g into 50 ml H2O, when the pellets have dissolved completely, adjust the volume to 100 ml), store at room temperature.

*Procedure*

To induce an acute inflammatory response, 360 *Tg(mfap4:mCherry-F)* larvae are either amputated or not amputated [7,11]. Caudal fin fold amputation is performed on 3 days post-fertilization (dpf) larvae. MCherry-F is used for specific labeling of the macrophages.

1. First, larvae are anaesthetized in zebrafish water supplemented with tricaine (final concentration 0.016% tricaine (MS-222)). For example, 60 larvae are placed into a petri dish filled with 25 ml zebrafish water (without methylene blue), then 400 µl of anesthesia tricaine (tricaine stock (4 mg/ml)) is added directly into the dish.
2. After approximatively 10 min, place the petri dish under a dissecting stereomicroscope, and manipulate the larvae in the center of the dish using a brush or a thin tip with an orientation allowing a lateral view.
3. Perform an amputation of the tip of caudal fin fold using a sterile scalpel blade, just posterior to the tip of the notochord with slight pressure, without hurting the notochord (Fig. 1), as described in [7].

**Fig. 1:**
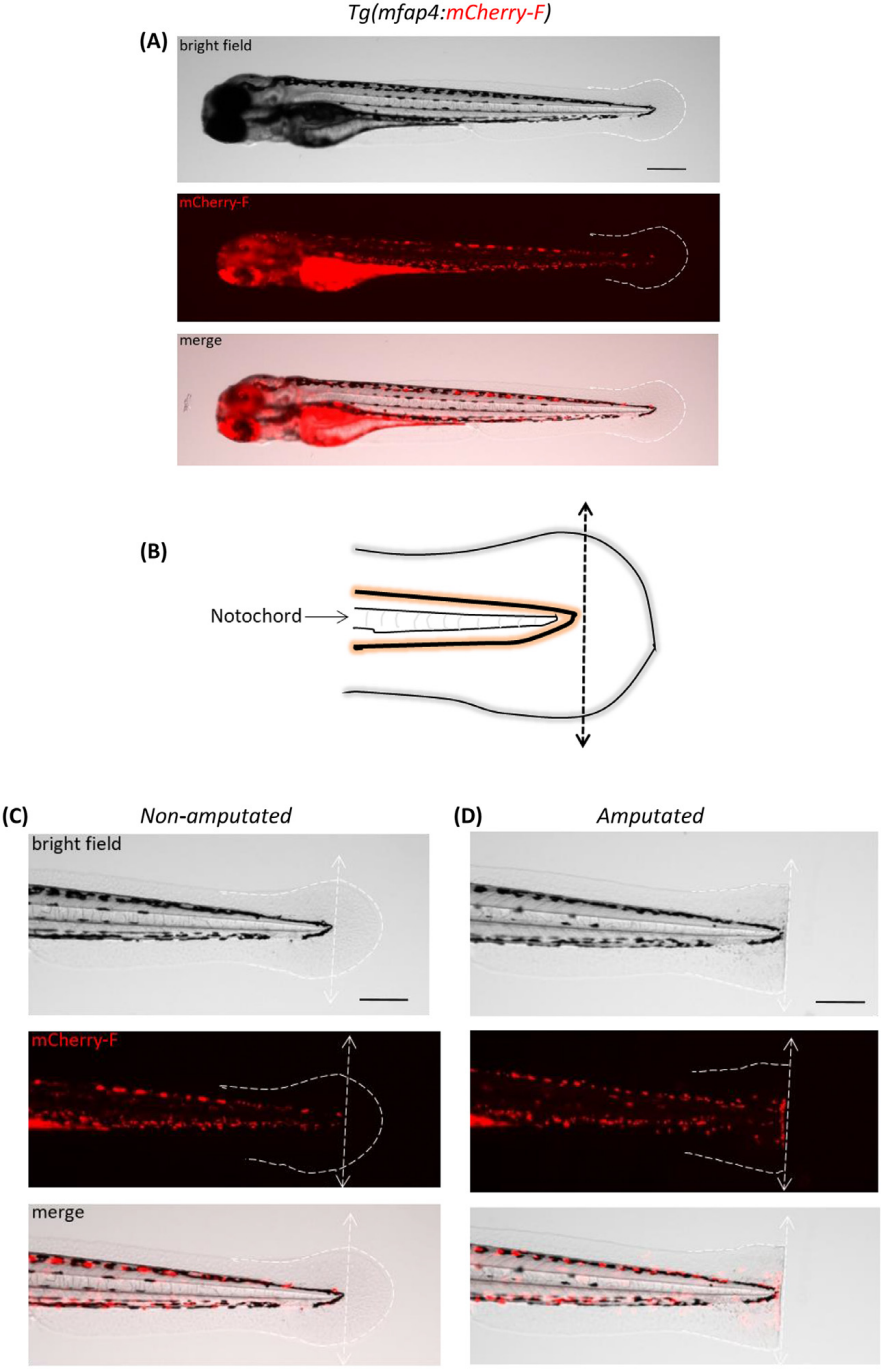
**Zebrafish larvae caudal fin fold amputation.** (**A**) Representative images of a *Tg(mfap4:mCherry-F)* larva at 3 days post-fertilization (3 dpf) show bright field, mCherry-F fluorescence and overlay. (**B**) Diagram showing caudal fin fold amputation without notochord damage; the amputation site is shown by the black arrow. (**C**) Higher magnification of a non-amputated fin fold and amputated fin fold. Representative images (bright field, mCherry-F fluorescence and overlay) show the absence of macrophages (mCherry-F fluorescence) in the non-amputated fin fold and (**D**) the recruitment of macrophages (mCherry-F fluorescence) at the wound in amputated fin fold. The white dashed line outlines the fin fold and the white arrow shows the amputation site. In (A), (B), (C) et (D) Scale bar: 200 µm.
4. Following amputation, with a transfer plastic pipet, transfer the sedated amputated larvae into a new petri dish filled with fresh tricaine free zebrafish water, to allow recovering of the larvae at 28 °C until the desired time points post-amputation.

#### Enzyme-free cell dissociation from whole wild-type AB and *Tg(mfap4:mCherry-F)* zebrafish larvae for FACS sorting (step2)

*Notes* ***:***

• *It has been shown by Nguyen-Chi et al.* [5] *that the use of trypsin for cell dissociation induces high level of pro-inflammatory genes, so do not use trypsin for inflammations studies*
• *0.9X DPBS is preferable than 1X DPBS for zebrafish cell viability*
• *Work as quickly as possible and maintain working reagents and macrophage suspension at 4* °*C*
• *Control wild-type zebrafish larvae should be prepared in the same way as the Tg(mfap4:mCherry-F) zebrafish larvae*
• *Do not add more than 150 whole larvae per cell strainer 70-µm mesh*

*Equipment*

- Ice box
- Cooling centrifuge for 50 ml tubes
- 50 ml centrifuge tubes (Corning # 430829)
- Cell Strainers 40-µm mesh, blue, sterile, individually (Falcon™ # 352340)
- Cell Strainers 70-µm mesh, white, sterile, individually (Falcon™ # 352350)
- 2.5 ml Syringe plunger (Terumo # SS*02SE1)
- 5 ml round-bottom polystyrene FACS tubes (Falcon™ # 352052)
- Generic laboratory equipment

*Reagents*

- 1X Dulbecco's Phosphate-Buffered Saline (1X DPBS), no calcium, no magnesium (Life Technologies # 14190094)
- Ultrapure 0.5 M EDTA, pH 8.0 (Invitrogen™ # 15575–020)
- **F**etal **B**ovine **S**erum, qualified, heat **i**nactivated (**FBSi** Gibco™ # 10500064)
- Working buffer (at 4 °C): 500 ml 0.9X DPBS with 2% FBSi and 2 mM EDTA

*Procedure*

1. Place a cell strainer 70-µm mesh on a 50 ml Falcon tube (Fig. 2A). With a 25 ml pipet, transfer anaesthetized larvae contained in two petri dishes (120 larvae) on the cell strainer 70-µm mesh (Fig. 2B).

**Fig. 2:**
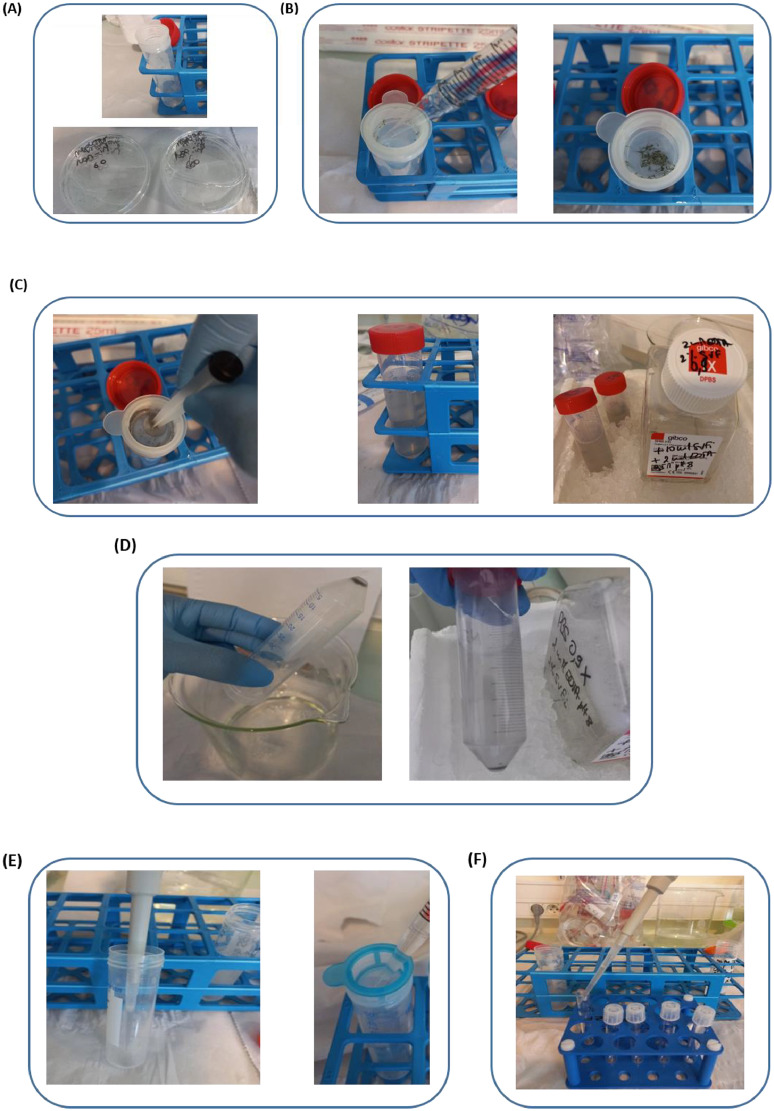
**Enzyme free cell dissociation from whole zebrafish larvae.** (**A**) Two petri dishes (120 larvae) for one cell strainer 70-µm mesh. (**B**) Transfer 120 anaesthetized larvae with a 25 ml pipet in the cell strainers 70-µm mesh. (**C**) With a 2.5 syringe plunger, crush larvae and rinse the filter with 30 to 50 ml of cold working buffer (0.9X DPBS with 2% FBSi and 2 mM EDTA). (**D**) After centrifugation (1700 rpm, 10 min, 4 °C), keep the 50 ml Falcon on ice. Discard the supernatant by inverting the Falcon tube (the cell pellet sticks to the bottom). (**E**) Suspend the cell pellet in 1 ml of cold working buffer with P1000 pipet (pipetting up and down) and adjust up to 10 ml. Place on a new 50 ml Falcon tube one cell strainer 40-µm mesh and filter the 10 ml cells suspension. (**F**) After centrifugation, suspend the cells pellet with 1.5 ml of cold working buffer and transfer them into 5 ml round bottom polystyrene FACS tube.
2. Discard the flow through. Rinse larvae with approximatively 10 ml of cold working buffer and discard the flow through.
3. With a 2.5 ml syringe plunger, crush larvae directly and quickly on the cell strainer. Rinse the syringe plunger and the filter with approximatively 30 to 50 ml of cold working buffer (Fig. 2C). Repeat the same procedure for the rest of the larvae (for example, for 360 larvae, use three 50 ml Falcon tube with three cell strainer 70-µm mesh).
4. Quickly centrifuge cell suspension at 1700 rpm, 10 min, at 4 °C. After centrifugation, keep the 50 ml Falcon on ice. Discard the supernatant by inverting the Falcon tube (the cell pellet sticks at the bottom of the 50 ml Falcon) (Fig. 2D).
5. Re-suspend the cell pellet in 1 ml of cold working buffer with a P1000 pipet by pipetting up and down (Fig. 2E). When the pellet is fully suspended, add up to 10 ml of cold working buffer.
6. Place a cell strainer 40-µm mesh on a new 50 ml Falcon tube and filter the 10 ml cell suspension (Fig. 2E). Rinse the filter with approximatively 2 to 6 ml of cold working buffer. Repeat the same procedure with other cell suspensions. Subsequently, pool the filtered cell suspensions belonging to the same condition into a single 50 ml Falcon. Quickly centrifuge the pooled cell suspension at 1700 rpm, 10 min, at 4 °C. After centrifugation, keep the Falcon on ice.
7. Discard the supernatant by inverting the 50 ml Falcon tube (the cell pellet sticks to the bottom of the 50 ml Falcon). Re-suspend the cell pellet in 1.5 ml of cold working buffer and transfer it into 5 ml Round Bottom Polystyrene FACS tube (Fig. 2F).
8. Keep the sample on ice until FACSAria cell sorting.
9. Proceed to cell sorting as quickly as possible.

#### Isolation of mCherry-F positive / mCherry-F negative cells using fluorescence activated cell sorting (BD FACSAria^TM^ IIu) (step3)

*Notes:*

• *A typical sorted sample would be performed in 30* min *maximum. Do not hesitate to change collecting tube every 30* min *for the same sorting sample*
• *If the sample does not pass through the FACSAria sample line, do not hesitate to filter the cell suspension through a cell strainer 40-µm mesh to ovoid clogged nozzle*
• *We strongly suggest to use a dead cell exclusion dye, for a cleaner separation and identification of cell populations*
• *An important step is to collect directly into the lysis buffer when the cell number is low (< 100,000 cells)*

*Equipment*

- BD FACSAria™ IIu
- RNase/DNase-free 1.5 ml microfuge tubes (Eppendorf # 0030 125.215)
- Cell Strainers 40-µm mesh, blue, sterile, individually (Falcon™ # 352340)
- Vortex agitator
- Generic laboratory equipment

*Reagents*

- RLT Buffer (provide Qiagen RNeasy micro Kit)
- β-Mercaptoethanol (β-ME Sigma # 60–24–2)
- RLT/ β-ME Lysis buffer (Add 10 µl β-ME to 1 ml RLT buffer Qiagen)
- SYTOX™ Red Dead Cell Stain, for 633 or 635 nm excitation (Invitrogen # S34859)

*Procedure*

**Fig. 3:**
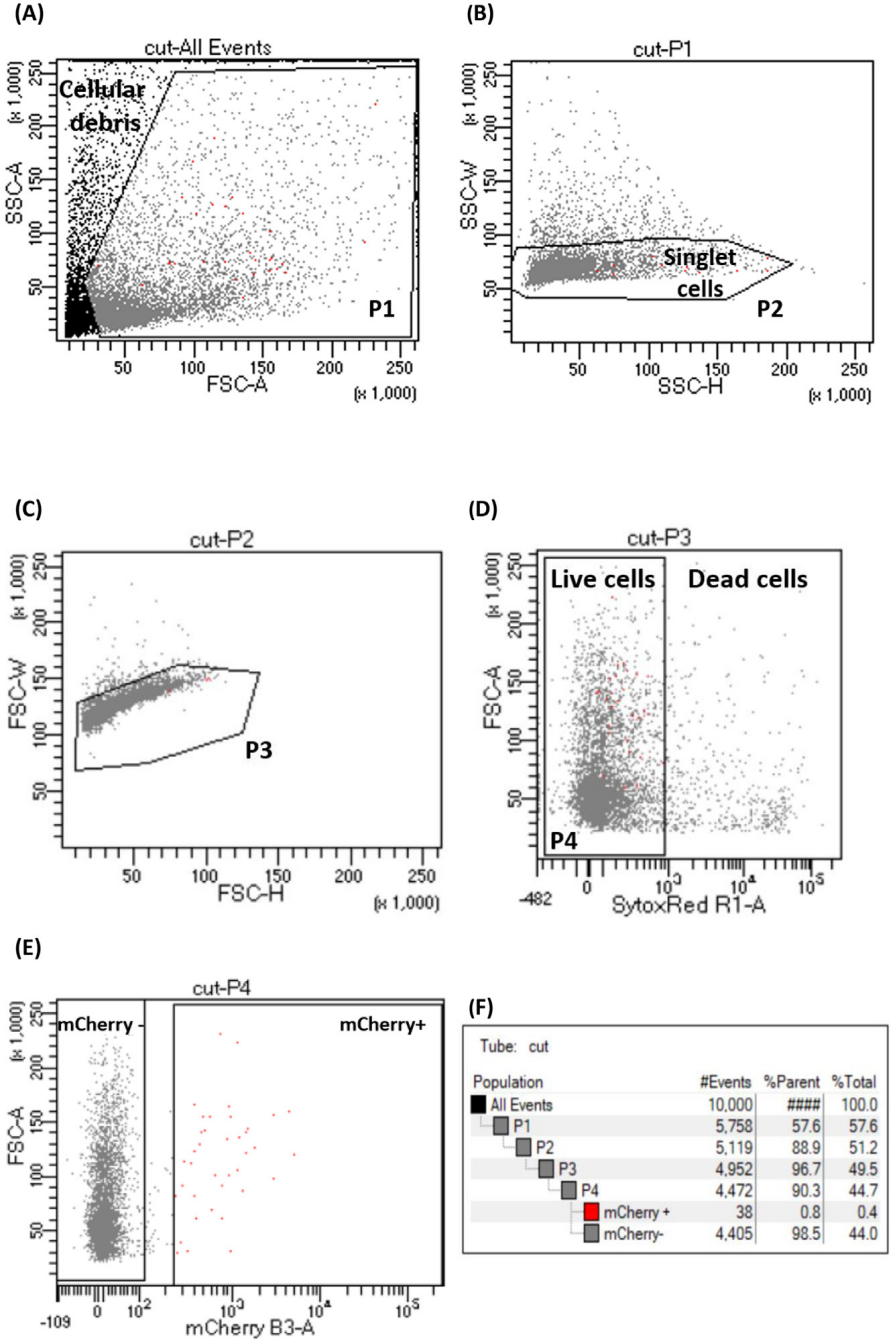
**Isolation of mCherry-F positive and mCherry-F negative macrophages from zebrafish larvae using Fluorescence Activated Cell Sorting (BD FACSAria™ IIu).** Flow cytometric plots show the sorting/gating strategy of live mCherry-F positive and mCherry-F negative macrophages. (BD FACSAria™ IIu, nozzle size: 70 µm, frequency: 87 kHz, sheath pressure: 70 psi, sheath fluid is 1X DPBS (No calcium)). ***S****ide****SC****atter****A****rea (SSC-A); Forward****SC****atter****A****rea (FSC-A);****S****ide****SC****atter****W****idth (SSC-W);****S****ide****SC****atter****H****eight (SSC—H)*; SytoxRed Area (SytoxRed R1-A); mCherry Area (mCherry B3-A). ***P****opulation (P) P1 to P4 represent the sub-populations starting from (****A****) all events without cellular debris (gate P1); (****B****) and (****C****) without doublet cells on SSC (gate P2) and on FSC (gate P3); (****D****) live cells (gate P4) and (****E****) mCherry-F negative (mCherry-) and mCherry-F positive (mCherry+) macrophages. (****F****) Gating strategy table with numbers of cells per gate (#Events), the percentage of cells in the parental gate (%Parent) and in relation to the total percentage of cells (%Total).*

1. In 1.5 ml of cell suspension from wild type line (with unlabeled macrophages), add 1 µl of 5 µM SYTOX™ Red Dead Cell Stain (final concentration 5 nM). This suspension is used for the cell viability and the gating strategy.
2. For cell sorting, use 1.5 ml of cell suspension from *Tg(mfap4:mCherry-F)* larvae. Macrophages are sorted according to their viability and red fluorescent signal using a BD FACSAria™ IIu cell sorter. Used SYTOX™ Red Dead Cell Stain at final concentration 5 nM (initial concentration = 5 µM) to allow the removal of dying or dead cells from sample. For example, add 1 µl into 1 ml cell suspension before cell sorting on FACSAria™ IIu.
3. The protocol described here uses an ARIA IIu (BD Biosciences, USA) driven by the BDFacsDiva 9.0.1 software.
4. The cell sorting is performed as following: Typical settings of the FACSAria™ IIu are: Nozzle size: 70 µm, Frequency: 87 kHz, sheath pressure: 70 psi, sheath fluid is 1X DPBS (No calcium).
5. Detection of fluorescence: we used a 488 nm laser line (100 mW) for excitation of the mCherry-F. The fluorescence is collected through 600 LP then 610/20 BP filters. SYTOX™ Red Dead Cell Stain is excited by a 633 nm laser line (30 mW). The emitted fluorescence is collected with a 660/20BP filter.
6. A refrigerated chamber keeps the sample at 4 °C and is agitated at 300 rpm. The flow rate is maintained between 3 and 5 to analyze 5000–6000 events per second and the sort efficiency rate is between 90 and 100%.
7. The settings for the sorting were carefully determined (Fig. 3) such as:

 Fig. 3. **A**: The dot plot (cut-All Events) shows P1 gate (**P**opulation **1**) which is used to exclude cellular debris and a part of dead cells. The sorting was made after selecting the cells of interest according to cell size (**f**orward **sc**atter **a**rea, FSC-A) versus granularity (**s**ide **sc**atter **a**rea, SSC-A)
 Fig. 3. **B**: The dot plot (cut-P1) shows P2 gate (**P**opulation **2**; singlet gating) which is used to exclude the doublet cells to keep only singlet cells according to **s**ide **sc**atter **w**idth (SSC-W) versus **s**ide **sc**atter **h**eight (SSC—H)
 Fig. 3. **C**: The dot plot (cut-P2) shows P3 gate (**P**opulation **3**) which is used to see only
 Singlet cells according to FSC-W versus FSC—H
 Fig. 3. **D**: The dot plot (cut-P3) shows P4 gate (**P**opulation **4**), which represents the selection of live cells based on SYTOX™ Red staining considering the singlet cells
 Fig. 3. **E**: The dot plot (cut-P4) shows macrophages that are sorted from the living cell population. The two gates show the selection of mCherry-F negative and positive cells based on their red fluorescence
 Fig. 3. **F**: Gating strategy table shows the numbers of cells per gate (#Events), the percentage of cells in the parental gate (%Parent) and the total percentage of cells (%Total).

8. To minimize RNA degradation and loss of material, the mCherry-F positive and mCherry-F negative sorted cells (from amputated (cut) and non-amputated (uncut) *Tg(mfap4:mCherry-F*) larvae) were separately collected directly into 100 µl RLT/β-ME Qiagen's lysis buffer into RNase/DNase-free 1.5 ml microfuge tubes.
9. After sorting, correctly close the cap of the collecting tube and vortex vigorously (2 × 5 s). Put the tube quickly on ice to process total RNA extraction immediately with RNeasy micro kit. Alternatively, tubes can be stored at - 80 °C for a long-term storage, until extraction of total RNA with RNeasy micro kit.

#### Total RNA extraction from a small number of mCherry-F positive sorted macrophages from amputated and non-amputated larvae (step4)

*Notes:*

• *Work as quickly as possible*
• *Follow precautions for handling RNA (for example, refer to precautions for handling of RNA Roche LifeScience)*
• *Treat the columns with DNase I (provided in kit)*
• *To use purified RNA for RNA sequencing or oligo-dt based amplification, do not add carrier RNA*
• *Be careful that the column does not contact the flow-through*
• *Eluate with 14 µl RNase-free water into RNase /DNase-free 1.5* *ml microfuge tubes*
• *After elution, immediately chill tubes on ice; keep 1.5 µl RNA into RNase/DNase-free 0.5* *ml microfuge tube for qualitative assessment*

*Equipment*

- A specific workbench for RNA preparation with generic laboratory equipment and respecting precautions for RNA handling
- Vortex agitator
- Microcentrifuge for 1.5 ml microfuge tubes
- RNase/DNase-free 1.5 ml microfuge tubes (Eppendorf # 0030 125.215)
- RNase/DNase-free 0.5 ml microfuge tubes (Eppendorf # 0030 121.023)

*Reagents*

- Qiagen RNeasy micro Kit (Cat. No. # 74004)
- Ultrapure DNase/RNase-free distilled water (Invitrogen # 10977035)
- 70% ethanol (Ethanol Absolute # 20821.310 and DNase/RNase-free water)

*Procedure*

1. After cell sorting, or thawing from −80 °C, lysates are kept on ice.
2. Vortex vigorously and with a pipet with sterile tip, measure precisely the exact final volume of sorted cells mixed with the RLT/βME Qiagen's Buffer.
3. According to the manufacturer's instructions, add exactly the same volume of 70% ethanol to the final volume of sorted sample, and mix immediately. As an example, 57,000 mCherry-F positive macrophages sorted into 100 µl RLT/βME Qiagen's buffer lead to a final volume of 200 µl; the total volume of 70% ethanol that needs to be added is therefore 200 µl.
4. Follow the Qiagen RNeasy micro Kit procedure according to the manufacturer's instructions.
5. Spare 1.5 µl of each RNA sample into a new RNase/DNase-free 0.5 ml microfuge tubes that will be used for the qualitative assessment of total RNA using the Bioanalyzer 2100.
6. Keep the rest of RNA samples at −20 °C for a short-term storage or at −80 °C for a long-term storage.

#### Qualitative assessment of total RNA (step5)

*Notes:*

• *Intact RNA is a key element for successful transcriptomic analysis*
• *Since a NanoDropTM is not appropriate to measure such low RNA concentrations, rather use Qubit® Fluorometer (with the RNA HS Assay Kits for example)*
• *To assess RNA integrity use Agilent 2100 Bioanalyzer*
• *Follow precautions for handling RNA (for example, refer to precautions for handling of RNA Roche LifeScience)*

*Equipment*

**Fig. 4:**
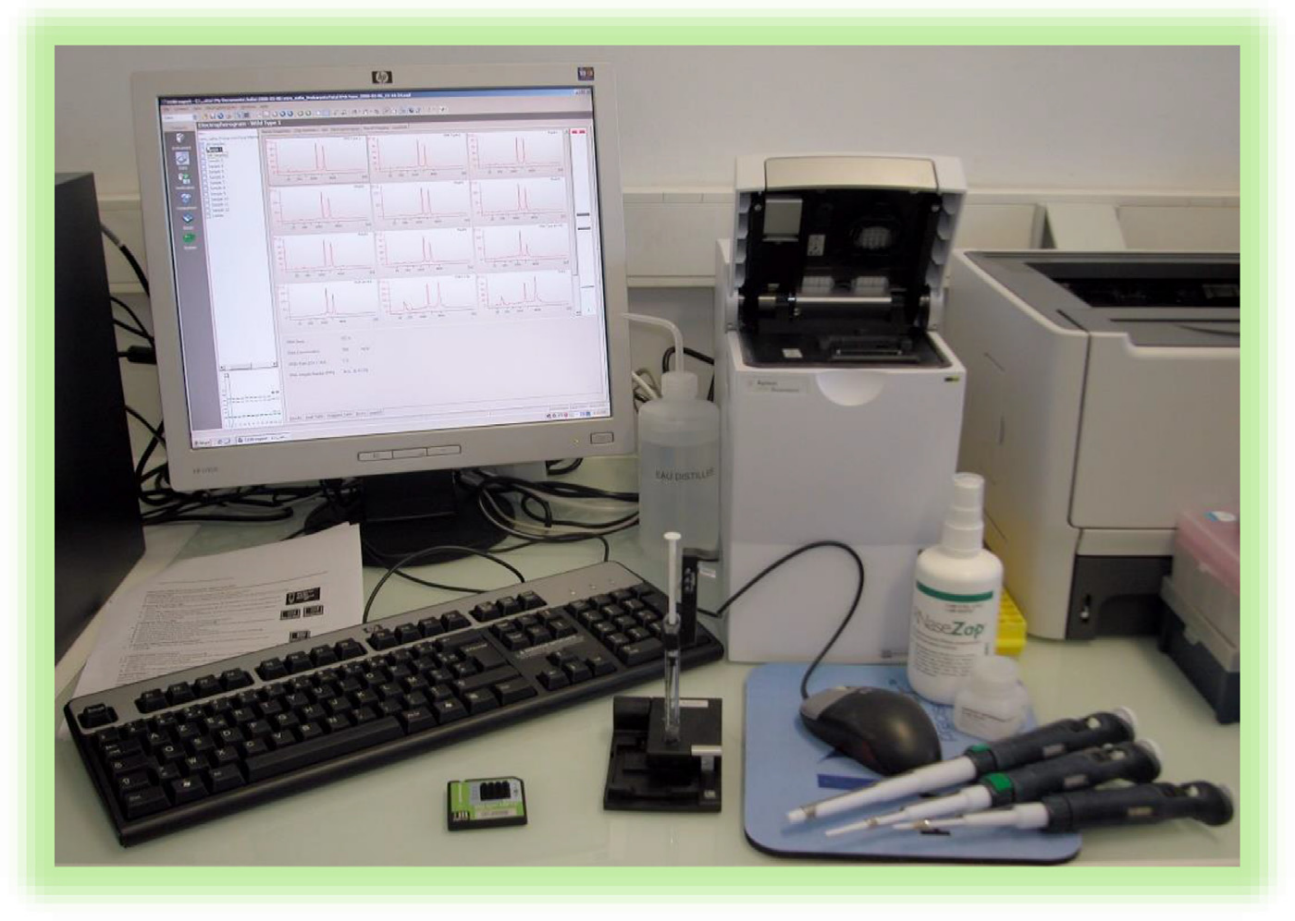
**Reagents and equipments for qualitative assessment of total RNA using the RNA 6000 Pico Kit and the Agilent 2100 Bioanalyzer Instrument.** Kit RNA 6000 Pico Bioanalyzer High Sensitivity RNA Analysis (Agilent # 5067–1513) contains chips and reagents designed for analysis of RNA fragments. Equipments supplied with the Agilent 2100 Bioanalyzer System are chip priming station (#5065–4401), IKA vortex mixer (#MS2-S8 or MS2-S9) and the Bioanalyzer Software (#G2946CA).
- Equipment supplied with the Agilent 2100 Bioanalyzer are chip priming station (#5065–4401) and IKA vortex mixer (#MS2-S8 or #MS2-S9)

- 2100 Bioanalyzer Instrument (Agilent # G2939BA) and its software (2100 Expert Software #G2946CA) (Fig. 4)

*Reagents*

- Kit RNA 6000 Pico Bioanalyzer High Sensitivity RNA Analysis (Agilent # 5067–1513)

*Procedure*

1. Thaw total RNA on ice (use the 1.5 µl RNA samples previously set aside).
2. Follow the RNA 6000 Pico Bioanalyzer Kit procedure according to the manufacturer's instructions.

### Data measurement and analysis

**Fig. 5:**
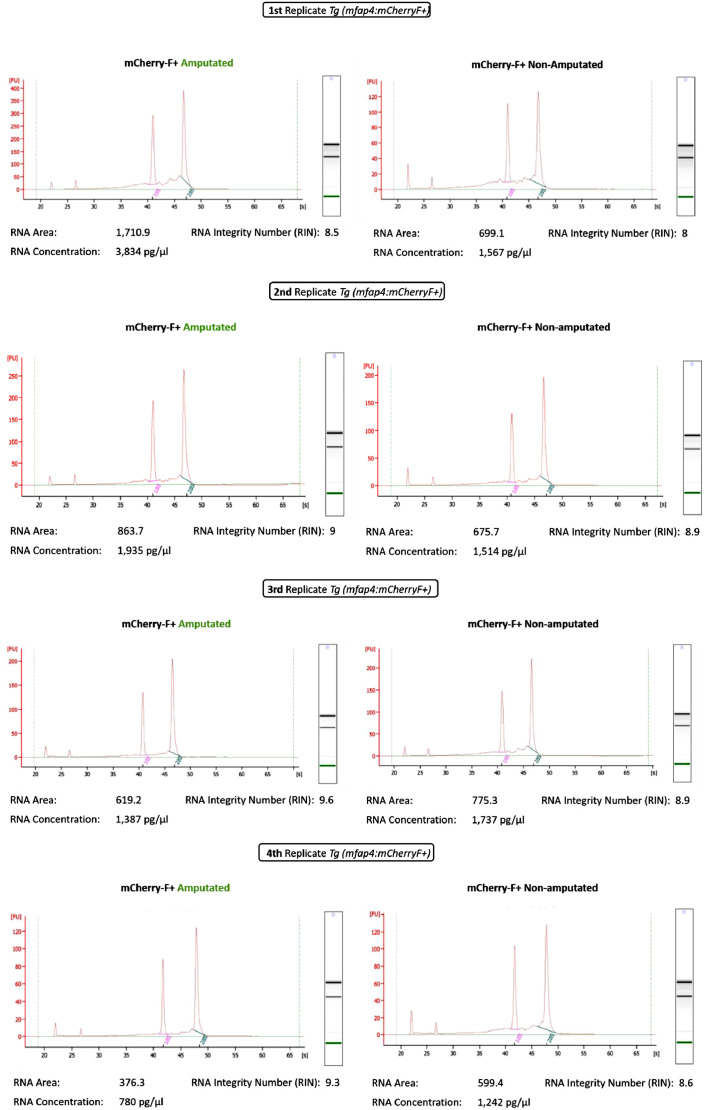
**Analysis of RNA integrity using the Agilent 2100 Bioanalyzer. Assessment of the quality of the extracted total RNAs using Pico gels and the Agilent 2100 Bioanalyzer.** RNA Integrity Number (RIN) are calculated based on electrophoresis plots using the 2100 Expert software. RIN were calculated from 4 independent experiments; they range from 8 to 9.6.

Here the Table 1 and Fig. 5 provide data that validate the protocol.

**Table 1:** **Representative number of sorted-mCherry-F positive macrophages, total RNA concentrations and total amount of RNA.** RNAs were prepared from sorted-mCherry-F positive macrophages from two conditions: amputated fin fold and non-amputated fin fold. The experiments were performed in 4 independent replicates. The 4 independent replicates (amputated and non-amputated) show that the total amount of RNA ranges from 15 to 50 nanograms for a number of mCherry-F positive macrophages ranging from 34,000 to 97,000 cells.

*Tg(mfap4: mCherryF)*	Conditions	Number of mCherryF+ macrophages cell sorted	Concentration of RNA (picog/µl)	Total amount of RNA in ng (into 14 µl RNAse-free water)
1st Replicate	Amputated	75,000	3834	54
	Non-amputated	34,655	1567	22
2nd Replicate	Amputated	70,268	1935	27
	Non-amputated	46,268	1514	21
3rd Replicate	Amputated	97,297	1387	19
	Non-amputated	67,397	1737	24
4th Replicate	Amputated	57,583	780	15
	Non-amputated	40,110	1242	17

The Table 1 recapitulates the number of mCherry-F positive macrophages that were sorted using the FACSAria™ IIu in the different conditions (amputated and non-amputated). The Table 1 also shows representative concentrations and total amount of total RNA. In the course of these 4 independent replicates (amputated and non-amputated), we sorted from 34,000 to 97,000 mCherry-F positive macrophages and obtained from 15 to 50 nanograms of total RNA. The Fig. 5 shows assessment of the integrity of the extracted total RNAs. RNAs were ran on Pico gels and analyzed using the Agilent 2100 Bioanalyzer. Sample integrity of RNA can be determined using the RNA Integrity Number (RIN). RIN were calculated from 4 independent experiments and the corresponding electrophoregrams reveal that RINs range from 8 to 9.6. These values support the use of this protocol to obtain RNA with good integrity from a small number of FACS-sorted mCherry-F positive macrophages, allowing us to continue with transcriptomic analysis thanks to the good RNA quality control.

## Conclusion

During inflammation, macrophage activation is associated with deep changes in their transcriptional profile. The zebrafish larva is becoming a popular model to study leukocytes thanks to its transparency and genetic tractability. Transcriptional profiling of macrophages in zebrafish larvae provided a valuable data resource to understand how macrophage respond to infection and inflammation [10]. However, this kind of analysis can be very challenging because the total number of macrophages per larva is low and it is difficult to obtain a high RNA integrity from a small number of isolated cells. Our objective was to optimize a protocol to obtain RNAs with a good integrity, in order to study the transcriptional programs of macrophages during inflammation in the zebrafish model. This protocol was optimized based on two publications: [6,5]. Contrarily to most protocols to dissociate larvae that use enzymes (collagenase, trypsin) [4,2,1], we developed an enzyme free protocol, combined with an osmotically controlled buffer (0.9X DPBS) to avoid over production of inflammatory mediators. In addition, our protocol was designed to maximize the total amount of extracted RNA and to favor good RNA quality. In addition, we demonstrated that this protocol is efficient to prepare RNAs from a small number of FACS-sorted zebrafish macrophages.

We believe that this protocol will be useful for all those interested in preparing RNAs with high RNA integrity from a small number of isolated macrophages to analyze macrophage transcriptional profiles in zebrafish.

## Funding

This work was supported by a grant from the European Community's H2020 Program [Marie-Curie Innovative Training Network Inflanet: Grant Agreement n° 955576], by the french Agence Nationale de la Recherche [ANR-19-CE15–0005–01, MacrophageDynamics], by Région Occitanie [Région Repere-Inflanet n° 21018327]. Funding sources had no role in the writing of the manuscript or the decision to submit it for publication.

## CRediT authorship contribution statement

**Christina Begon-Pescia:** Methodology, Data curation, Writing - original draft, Writing - review & editing. **Stéphanie Boireau:** Data acquisition, Writing - review & editing. **Myriam Boyer-Clavel:** Data acquisition, Writing - review & editing. **Georges Lutfalla:** Visualization, Supervision, Methodology, Data curation, Writing - original draft, Writing - review & editing. **Mai Nguyen-Chi:** Visualization, Supervision, Methodology, Data curation, Writing - original draft, Writing - review & editing.

## Declaration of Competing Interest

The authors declare that they have no known competing financial interests or personal relationships that could have appeared to influence the work reported in this paper.
